# Influence of Hypertension on the Regional Elastic Properties of Ascending Thoracic Aortic Aneurysms with Bicuspid Aortic Valves

**DOI:** 10.1007/s10439-026-04101-0

**Published:** 2026-04-16

**Authors:** Yue Yu, Yuan-Feng Xin, Fan Yang, Xiaojuan Xu, Gerhard A. Holzapfel, Jianhua Tong

**Affiliations:** 1 Institute for Biomedical Engineering and Nano Science, Shanghai East Hospital, Tongji University School of Medicine, 500 Zhennan Road, Shanghai, 200331 P.R. China; 2 Department of Cardiovascular Surgery, Shanghai East Hospital, Tongji University School of Medicine, Shanghai, P.R. China; 3 School of Aerospace Engineering and Applied Mechanics, Tongji University, Shanghai, P.R. China; 4 Institute of Biomechanics, Graz University of Technology, Stremayrgasse 16/2, 8010 Graz, Austria; 5 Department of Structural Engineering, Norwegian University of Science and Technology (NTNU), Trondheim, Norway

**Keywords:** Ascending thoracic aortic aneurysm, Failure property, Biaxial extension test, Hypertension

## Abstract

Although hypertension may affect the local biomechanical properties of ascending thoracic aortic aneurysms (ATAAs) with a bicuspid aortic valve (BAV), a comparison of the regional elastic properties of BAV-ATAAs between patients with and without hypertension is not yet available due to lack of biomechanical data. In this study, we collected 25 fresh ATAA samples from 17 hypertensive and 8 age-matched non-hypertensive patients who underwent elective aortic surgery. Biomechanical tests were executed to investigate the regional failure stresses and biaxial mechanical properties of BAV-ATAAs with and without hypertension. A material model was fitted to the biaxial experimental data to obtain model parameters in different regions. Histological analysis was performed to investigate the underlying microstructure and determine the percentages of elastic and collagen fibers. The circumferential failure stresses in the anterior, lateral, and posterior regions were significantly lower for the hypertensive group than in the non-hypertensive group. Regarding equibiaxial stresses, the hypertensive BAV-ATAAs showed significantly higher longitudinal tissue stiffness in the anterior and lateral regions than the non-hypertensive BAV-ATAAs. Collagen fiber hyperplasia was observed in the anterior and lateral regions of both the hypertensive and non-hypertensive BAV-ATAAs. However, in these regions the laminar structure of the elastic fibers was disrupted in several places and apoptotic smooth muscle cells were observed in the group with hypertension compared to the group without hypertension. In addition, the circumferential failure stresses in the anterior and lateral regions were significantly increased in the hypertensive group and strongly correlated with the collagen content. These results suggest that the regional elastic properties of the hypertensive BAV-ATAAs are more deteriorated than those of the non-hypertensive samples. Due to the significant impact on circumferential tensile strength, the hypertensive BAV patients may be at a higher risk of ATAA rupture than non-hypertensive BAV patients.

## Introduction

Ascending thoracic aortic aneurysm (ATAA) is a degenerative vascular disease characterized by an asymmetric bulge of the human ascending aorta [12]. Untreated ATAA may lead to life-threatening aortic events such as aortic dissection (AD) or aortic rupture, which are associated with a high mortality rate [26, 31]. Patient-specific biomechanical properties of ATAAs are becoming increasingly important to clarify the risk assessment of these complications and may help us better understand the underlying etiology [1, 9, 11, 23, 24, 42]. Notably, regional variations in biomechanical properties exist in the majority of dilated ascending aortas, regardless of aortic valve morphologies [7, 21, 43]. 

Both hypertension and the bicuspid aortic valve (BAV) are considered important clinical risk factors that may alter the local biomechanical properties of the ATAAs. Hypertension contributes significantly to structural remodeling of the ascending aorta [4, 27, 30], while the BAV is frequently associated with aneurysm formation due to blood circulatory disturbances [22, 26, 35, 41]. As mentioned previously, ATAA tissues showed a deterioration in their delamination and elastic properties when affected by either hypertension or BAV [1, 5, 13, 40, 42]. It should be emphasized that most BAV-ATAAs acquired during aortic surgical repair are associated with a history of hypertension [19, 26]. A recent study from our laboratory [43] suggests that hypertensive BAV-ATAAs exhibit pronounced regional heterogeneity in biaxial mechanical behavior. Due to a lack of biomechanical data of ATAA samples from age-matched non-hypertensive patients, the differences in the regional elastic properties of BAV-ATAAs between hypertensive and non-hypertensive groups are not yet well understood.

This study has three objectives: (i) to experimentally investigate the regional elastic properties of the hypertensive and non-hypertensive BAV-ATAAs; (ii) to determine regional biomechanical variables, including failure stress and aortic stiffness, for the hypertensive and non-hypertensive groups and to characterize the biaxial mechanical behavior of the BAV-ATAAs by material modeling; (iii) to investigate the underlying microstructural alterations in histology and to correlate biomechanical variables with histological characteristics.

## Materials and Methods

### Patient Population

Between March 2023 and July 2025, fresh ATAA samples (*n* = 25) were collected from 17 hypertensive and 8 non-hypertensive patients undergoing elective aortic surgery at Shanghai East Hospital. Patients with Marfan syndrome were excluded from the study. All patients in the hypertensive group had a history of hypertension before enrollment according to the latest European Society of Hypertension (ESH) guidelines. Moreover, these patients were taking antihypertensive drugs before aortic surgery to maintain their blood pressure within the normal range. The maximum ATAA diameter was calibrated using preoperative computed tomography (CT). Aortic valve morphotype was determined using echocardiography. After removal, the aortic samples with the entire circumferences were stored in phosphate-buffered saline (PBS) solution at 4 °C after harvesting and immediately transferred to the biomechanics laboratory for mechanical testing. This study was approved by the research ethics committee of Shanghai East Hospital of Tongji University School of Medicine in accordance with the Helsinki Declaration. All participants provided written informed consent.

### Specimen Preparation

**Fig. 1:**
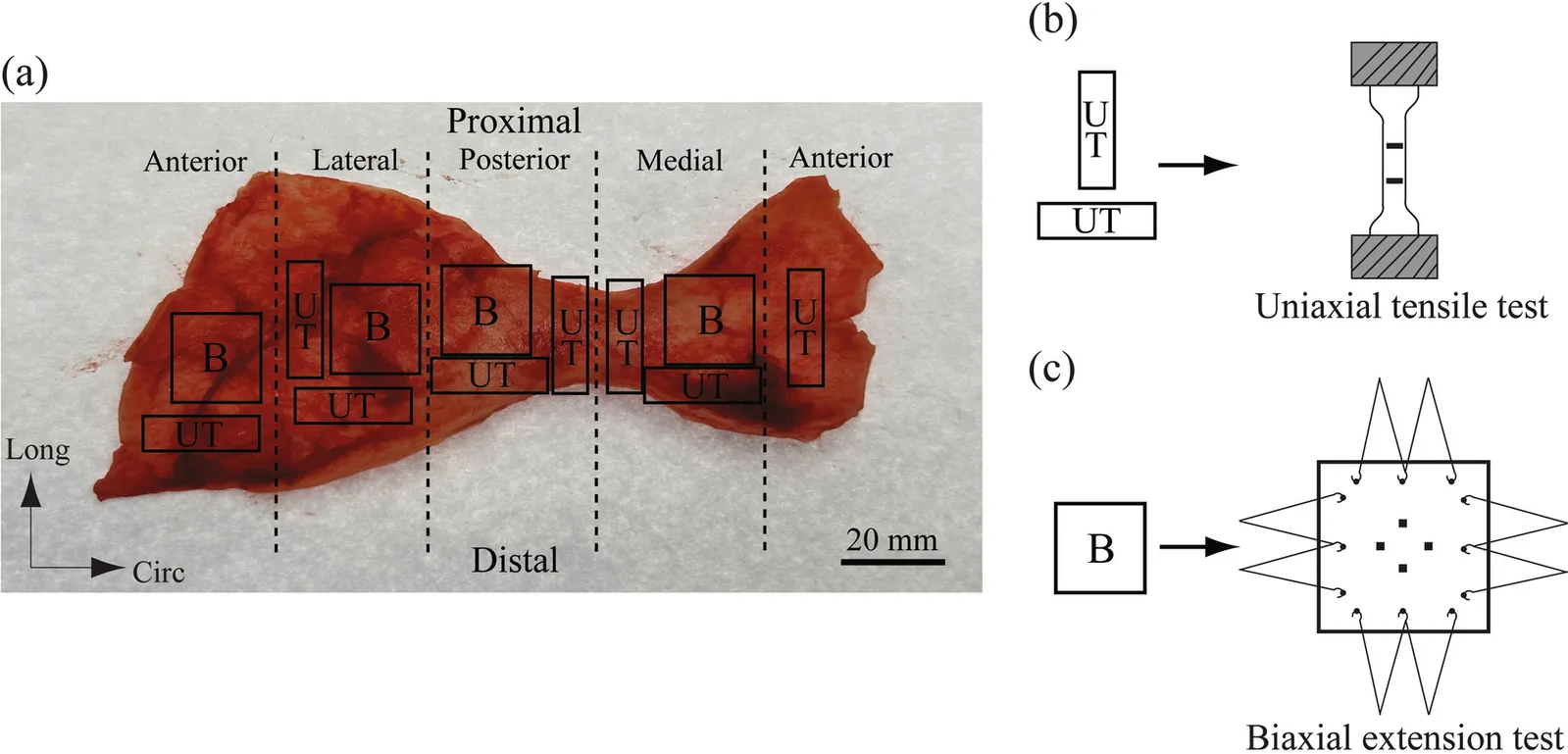
(**a**) Representative photograph of a complete fresh BAV-ATAA sample harvested from a non-hypertensive patient (age, 59 years) during elective aortic surgery. After open incision and cleaning, the anterior, lateral, posterior, and medial regions are sequentially divided by dashed lines according to their circumferential locations. For uniaxial tensile tests, rectangular strips (UT) are dissected from each region in the circumferential and longitudinal directions. For biaxial extension tests, square specimens (B) with a uniform dimension of 18 × 18 mm (length × width) are cut from each region. (**b**) To prepare for the UT test, a dog-bone shaped specimen was punched out from the rectangular UT strip. Sandpaper was mounted to both ends of the dog-bone shaped specimen, while two markers were glued to the middle part. (**c**) For the biaxial test, sutures were fixed to each edge of the square specimen with fishhooks, and four markers were placed in the center of specimen B. “Circ” and “Long” denote the circumferential and longitudinal directions, respectively

First, peripherally adherent, loose tissue was removed from the ascending aorta. As suggested by Lin et al. [21], the entire tissue piece was divided into anterior, lateral, posterior, and medial regions with respect to its circular location after an open incision (see Fig. 1a). Square specimens B measuring 18 × 18 mm were excised from each region for biaxial extension tests (Fig. 1a). In addition, rectangular strips measuring approximately 22 × 6 mm were cut for uniaxial tensile (UT) tests (Fig. 1a). Furthermore, a small piece of tissue was prepared from each region for histological analysis. The thickness of the specimen was measured using a video-extensometer [38, 42]. To prepare for the UT test, dog-bone shaped specimens were punched out of the rectangular strips. Sandpaper was mounted at both ends of the dog-bone shaped specimens, and two markers were glued to the middle part (Fig. 1b). For the biaxial test, sutures were attached with fishhooks to each edge of the square specimen, and four markers were placed in the center of the specimen (Fig. 1c). All specimens were moistened with PBS prior to testing to prevent drying, which could significantly alter the biomechanical properties of the aortic tissue.

### Biomechanical Testing

The UT test was carried out on a PC-controlled, high-precision tensile testing device for biological soft tissue (XJ810-10N, Xiangjie Inc., Shanghai, China). The tensile force was measured using a custom-built load cell with a maximum load capacity of 25 N, while the tensile stretch was tracked using a CCD camera. The entire test was conducted in a Plexiglas container filled with 0.9% PBS and maintained at 37.0 ± 0.1 °C. The specimens were preconditioned to 50 kPa in five loading and unloading cycles at the prescribed extension rate of 1 mm/min. Starting with the sixth cycle, the specimen was extended at the same extension rate until the tissue ruptured. The ultimate tensile forces and stretches were documented.

The methodology of biaxial testing was presented in detail in our previous studies [39, 43]. Briefly, square specimens were mounted in a custom-built biaxial testing device and immersed in a bath filled with Dulbecco’s Modified Eagle’s Medium at 37.0 ± 0.1 °C. The four load-cells (Model-1/25, Xiangjie Inc., Shanghai, China) were manufactured with a maximum load capacity of 25 N and a minimum load resolution of 1 mN. The initial gauge lengths between two markers were measured with a CCD camera before the start of the biaxial test, while the current gauge lengths between two markers were continuously recorded with a CCD camera during the loading cycle. A stress-driven protocol was adopted to perform the biaxial testing. Each specimen was tested at each stress level using the following protocol: *P*_*θθ*_ : *P*_*LL*_ = 1 : 1, 1 : 0.75, 0.75 : 1, 1: 0.5 and 0.5 : 1, where *P*_*θθ*_ and *P*_*LL*_ denote the applied engineering stresses in the circumferential and longitudinal directions, respectively. In each protocol, the ratio between the applied engineering stresses *P*_*θθ*_ : *P*_*LL*_ remained constant. We initially applied *P*_*θθ*_* = P*_*LL*_ = 60 kPa to the specimen to perform the biaxial protocol and then gradually increased the stress by 20 kPa until rupture occurred. A stress rate of 10 kPa/s was employed during the loading cycle. At each stress level, the specimen underwent five preconditioning cycles, and the sixth cycle was used for data analysis.

### Data Analysis

For the UT test, the Cauchy stresses *σ* were plotted as a function of stretches *λ*. The failure stress ${\sigma }_{\mathrm{ult}}$ of the tissue specimen was calculated by

1 $${\sigma }_{\mathrm{ult}}=\frac{{f}_{\mathrm{max}}{ \lambda }_{\mathrm{ult}}}{WT} ,$$

where *f*_max_ is the measured maximum force, *λ*_ult_ is the corresponding stretch, and *W* and *T* are the width and thickness of the specimen in the unloaded configuration, respectively.

With negligible shear deformation during biaxial extension, the Cauchy stresses ${\sigma }_{\theta \theta }$ and ${\sigma }_{LL}$ were determined as

2 $${\sigma }_{\theta \theta }=\frac{{f}_{\theta }{\lambda }_{\theta }}{T{X}_{L}}{, \sigma }_{LL}=\frac{{f}_{L}{\lambda }_{L}}{T{X}_{\theta }},$$

where *λ*_*θ*_ = *x*_*θ*_ /*X*_*θ*_ and *λ*_*L*_* = x*_*L*_* /X*_*L*_ denote the tissue stretches in each direction measured by markers, while *X*_*θ*_ and *X*_*L*_ are the initial dimensions of the square specimen in the circumferential and longitudinal directions, respectively. To be consistent with our previous study [43], 140 kPa was chosen to compare biaxial mechanical data between hypertensive and non-hypertensive groups. The maximum tangential modulus (MTM), defined as the slope of the nonlinear stress-stretch curve under the equibiaxial stresses of *P*_*θθ*_* = P*_*LL*_ = 140 kPa, was calculated and averaged to evaluate aortic stiffness in the circumferential and longitudinal directions.

### Material Modeling

The material model of Holzapfel et al. [18] was used to characterize the biaxial mechanical behaviors of the BAV-ATAAs. The strain-energy function reads

3 $$\psi = \mu \left( {I_{1} - 3} \right) + \frac{{k_{1} }}{{k_{2} }}\left( {\exp \left\{ {k_{2} \left[ {\left( {1 - \rho } \right)\left( {I_{1} - 3} \right)^{2} + \rho \left( {I_{4} - 1} \right)^{2} } \right]} \right\} - 1} \right)$$

where *μ* > 0 and *k*_1_ > 0 are stress-like parameters (in kPa), and *k*_2_ > 0 and *ρ* ∈ [0,1] are dimensionless parameters, where *ρ* is considered as a switch parameter between isotropy and anisotropy. The invariants *I*_1_ and *I*_4_ are expressed as

4 $$I_{1} = \lambda_{\theta }^{2} + \lambda_{L}^{2} + \lambda_{r}^{2} ,\,\,\,\,\,\,\,I_{4} = \lambda_{\theta }^{2} {\mathrm{cos}}^{2} \varphi + \lambda_{L}^{2} {\mathrm{sin}}^{2} \varphi ,$$

where* λ*_*θ*_, *λ*_*L*_, and *λ*_*r*_ are the principal stretches in the circumferential, longitudinal and radial directions, respectively. The geometrical parameter *φ* in Eq. (4) denoted the angle between the principal orientation of the collagen fibers and the circumferential direction. Here *φ* was treated as a phenomenological variable.

Based on the assumption of incompressibility *λ*_*r*_*λ*_*θ*_*λ*_*L*_ = 1, the Cauchy stresses in the circumferential and longitudinal directions were determined as

5 $$ \begin{aligned}   \sigma _{{\theta \theta }}^{\Psi }  &  = 2\left( {\lambda _{\theta }^{2}  - \lambda _{\theta }^{{ - 2}} \lambda _{L}^{{ - 2}} } \right)\Psi _{1}  + 2\lambda _{\theta }^{2} \cos ^{2} \varphi \Psi _{4} ,\\ &\ \sigma _{{LL}}^{\Psi }  = 2\left( {\lambda _{L}^{2}  - \lambda _{L}^{{ - 2}} \lambda _{\theta }^{{ - 2}} } \right)\Psi _{1}  + 2\lambda _{L}^{2} \sin ^{2} \varphi \Psi _{4}  \\  \end{aligned}  $$

where the abbreviations ${\Psi }_{i}= \partial \Psi /\partial {I}_{i}$, *i* = 1, 4, were used.

Experimental data from five biaxial protocols for each square specimen in the circumferential and longitudinal directions were simultaneously fitted to the material model (3) using the ‘lsqnonlin’ optimization toolbox in Matlab R2020b (MathWorks, USA). The fitting was executed following the nonlinear least-squares algorithm, which minimized the sum of squares of the deviation between the predicted stresses and the experimental stresses. The square of the Pearson’s correlation coefficient was computed to evaluate the ‘goodness of fit’.

### Histological Analysis

The specimens were fixed in 4% paraformaldehyde phosphate buffer solution and then embedded in paraffin. The paraffin blocks were cut into 5 μm thick sections along the circumferential direction and stained with Masson, Elastica van Gieson and TUNEL. Histological sections were examined under a Leica DM2500 microscope (Leica Microsystems, Germany) to identify morphological changes of elastic and collagen fibers and other cellular components in the hypertensive and non-hypertensive BAV-ATAAs. Eight locations were randomly chosen from each scanned slide and captured at 40 × magnification. The magnified images were separately analyzed by two pathologists in a blinded study. A semiquantitative measurement using ImageJ to determine the component percentages of elastic and collagen fibers.

### Statistics

Results were expressed as mean ± standard deviation (SD). The Shapiro-Wilk test was used to determine the normal distribution of the dataset. An analysis of variance (ANOVA) followed by the post-hoc Tukey test was performed to compare the biomechanical variables of failure stress and MTM between multiple regions. The unpaired *t*-test was performed to compare the variables between hypertensive and non-hypertensive groups within each region. A *p*-value of < 0.05 determined statistical significance. The SPSS statistical package version 21.0 (SPSS Inc./IBM, Chicago, USA) was used for all statistical analyses.

Regional failure stress and aortic stiffness were plotted as a function of collagen fraction for the hypertensive and non-hypertensive BAV-ATAAs. A linear regression model was used to fit the scattered data using the optimization toolbox of OriginPro 9.0 (OriginLab, USA). The Pearson’s rank correlation coefficient *R* and the Spearman rank order correlation coefficient were employed to evaluate correlation. A strong correlation was considered when *|R|* > 0.60. A *p*-value < 0.05 determined significance.

## Results

### Clinical Characteristics of Patient Population

A total of 17 hypertensive and 8 non-hypertensive age-matched BAV-ATAA samples were collected and analyzed. The clinical characteristics of the patient population are presented in Table 1. The diameters of the ascending aortic of the hypertensive and non-hypertensive BAV patients were 52.8 ± 4.7 mm and 47.5 ± 3.9 mm, respectively (*p* = 0.27). No significant differences were found between two groups regarding averaged wall thickness, body mass index, diabetes, and hyperlipidemia (*p* > 0.05). There were no significant differences in aortic stenosis or systolic/diastolic blood pressure range in the early follow-up period between patients with and without hypertension (*p* > 0.05).

**Table 1:** Clinical characteristics of patient groups with and without hypertension

Characteristic	Hypertensive (n = 17)	Non-hypertensive (n = 8)	*p* value
Age, years	60 ± 9	57 ± 7	0.47
Male, %	82.4	75	0.76
BMI	26.8 ± 4.2	25.9 ± 3.5	0.95
Ascending aortic diameter, mm	52.8 ± 4.7	47.5 ± 3.9	0.27
Averaged wall thickness, mm	2.4 ± 0.4	2.1 ± 0.3	0.33
RL cusp fusion, %	88.2	87.5	> 0.99
Diabetes, %	17.6	12.5	0.87
Hyperlipidemia, %	11.8	12.5	> 0.99
Aortic stenosis (>moderate), %	23.5	12.5	0.69
SBP/DBP in the early follow-up, mmHg	120-140/70-90	100-130/60-80	0.21
CCB + ARB/ACEI + β-blocker use, %	100	0	< 0.001

BMI, body mass index; RL, cusp fusion, right-left cusp fusion; SBP, systolic blood pressure; DBP, diastolic blood pressure; CCB, calcium channel blocker; ARB, angiotensin receptor blocker; ACEI, angiotension-converting enzyme inhibitor

### Failure Stress

**Fig. 2:**
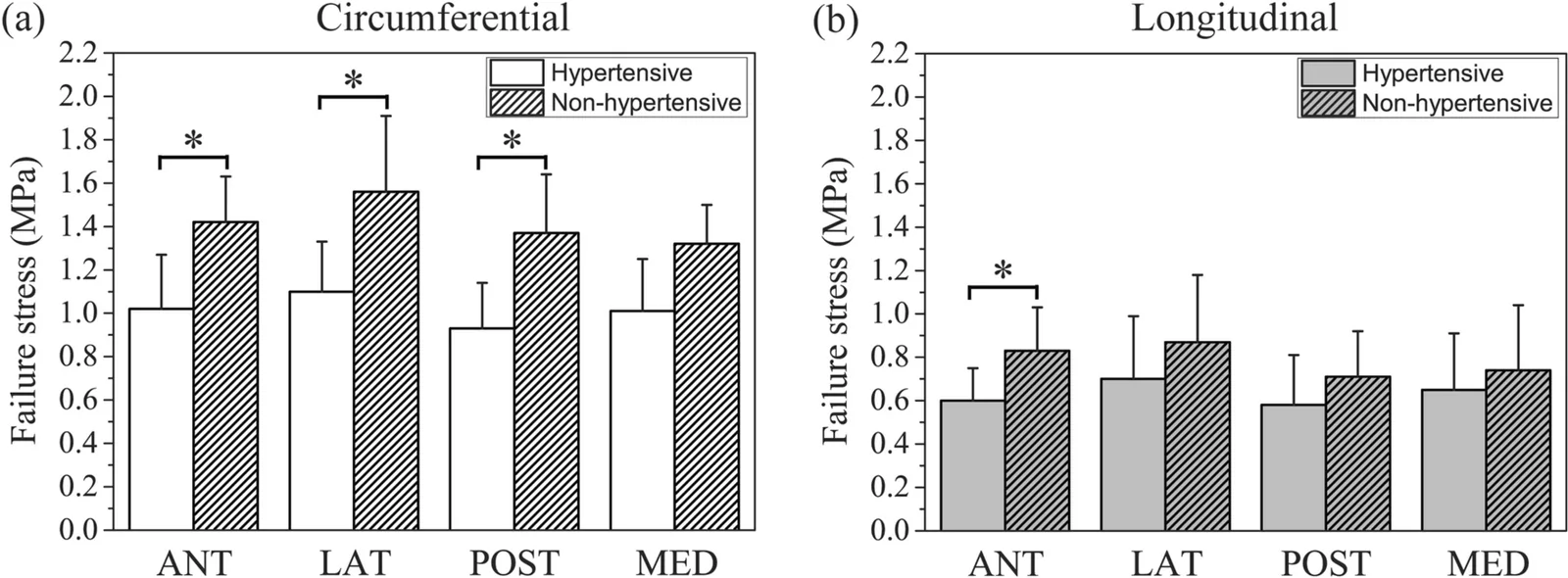
The computed circumferential and longitudinal failure stresses in four regions of the hypertensive and non-hypertensive BAV-ATAAs. ANT, LAT, POST, and MED denote the anterior, lateral, posterior, and medial regions, respectively. In the anterior, lateral, and posterior regions, the circumferential failure stresses are significantly lower in the hypertensive group than in the non-hypertensive group

The circumferential and longitudinal failure stresses in four different regions for hypertensive and non-hypertensive groups are shown in Fig. 2. The relevant quantitative values are summarized in Table 2. In the anterior, lateral, and posterior regions, the failure stresses in the circumferential direction were significantly lower for the hypertensive BAV-ATAAs than for the non-hypertensive BAV-ATAAs (1.02 ± 0.25 vs. 1.42 ± 0.21 MPa, *p* = 0.01; 1.1 ± 0.23 vs. 1.56 ± 0.35 MPa, *p* = 0.02; 0.93 ± 0.21 vs. 1.37 ± 0.27 MPa, *p* = 0.01). Remarkably, the longitudinal failure stresses in the anterior region of the hypertensive BAV-ATAAs were significantly lower than those of the non-hypertensive BAV-ATAAs (0.6 ± 0.15 vs. 0.83 ± 0.2 MPa, *p* = 0.04). In the lateral, posterior, and medial regions, there were no statistically significant differences in longitudinal failure stress between the hypertensive and non-hypertensive groups (*p* > 0.05). Furthermore, no statistically significant differences in ultimate stretches were found between the hypertensive and non-hypertensive groups in four different regions (*p* > 0.05).

**Table 2:** Circumferential and longitudinal failure stresses in the anterior, lateral, posterior, and medial regions of hypertensive and non-hypertensive BAV-ATAAs

		Failure stress ${\sigma }_{\mathrm{ult}}$ (MPa)		
		Hypertensive (*n* = 17)	Non-hypertensive (*n* = 8)	*p-*value
Anterior	Circ	1.02 ± 0.25	1.42 ± 0.21	0.01
	Long	0.6 ± 0.15	0.83 ± 0.2	0.04
Lateral	Circ	1.1 ± 0.23	1.56 ± 0.35	0.02
	Long	0.7 ± 0.29	0.87 ± 0.31	0.17
Posterior	Circ	0.93 ± 0.21	1.37 ± 0.27	0.01
	Long	0.58 ± 0.23	0.71 ± 0.21	0.19
Medial	Circ	1.01 ± 0.24	1.32 ± 0.18	0.12
	Long	0.65 ± 0.26	0.74 ± 0.3	0.33

### Biaxial Mechanical Properties

**Fig. 3:**
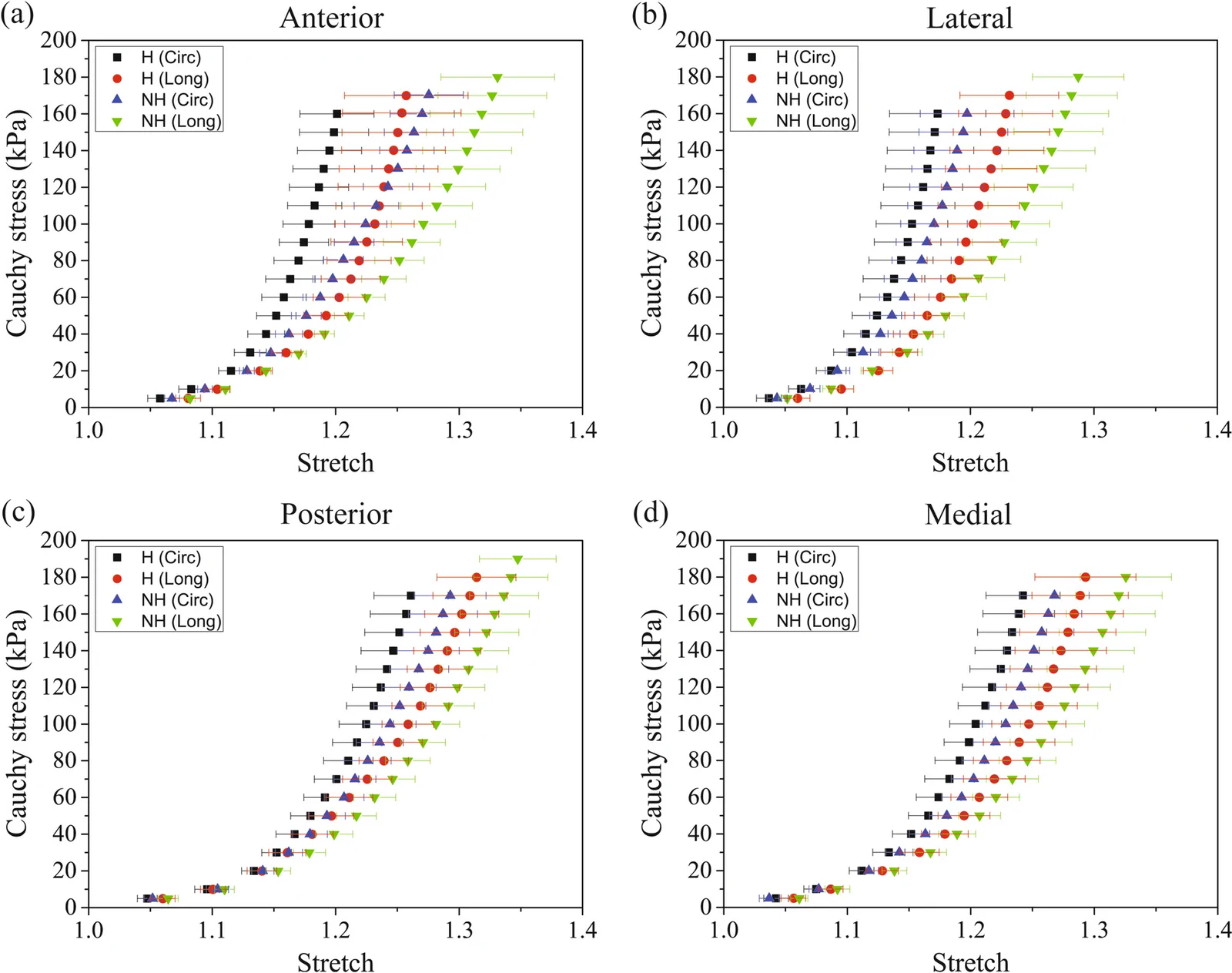
Averaged circumferential and longitudinal Cauchy stress versus stretch curves at equibiaxial stresses (*P*_*θθ*_* = P*_*LL*_ = 140 kPa) in four regions of hypertensive (H) and non-hypertensive (NH) BAV-ATAAs. “Circ” and “Long” denote the circumferential and longitudinal directions, respectively

**Fig. 4:**
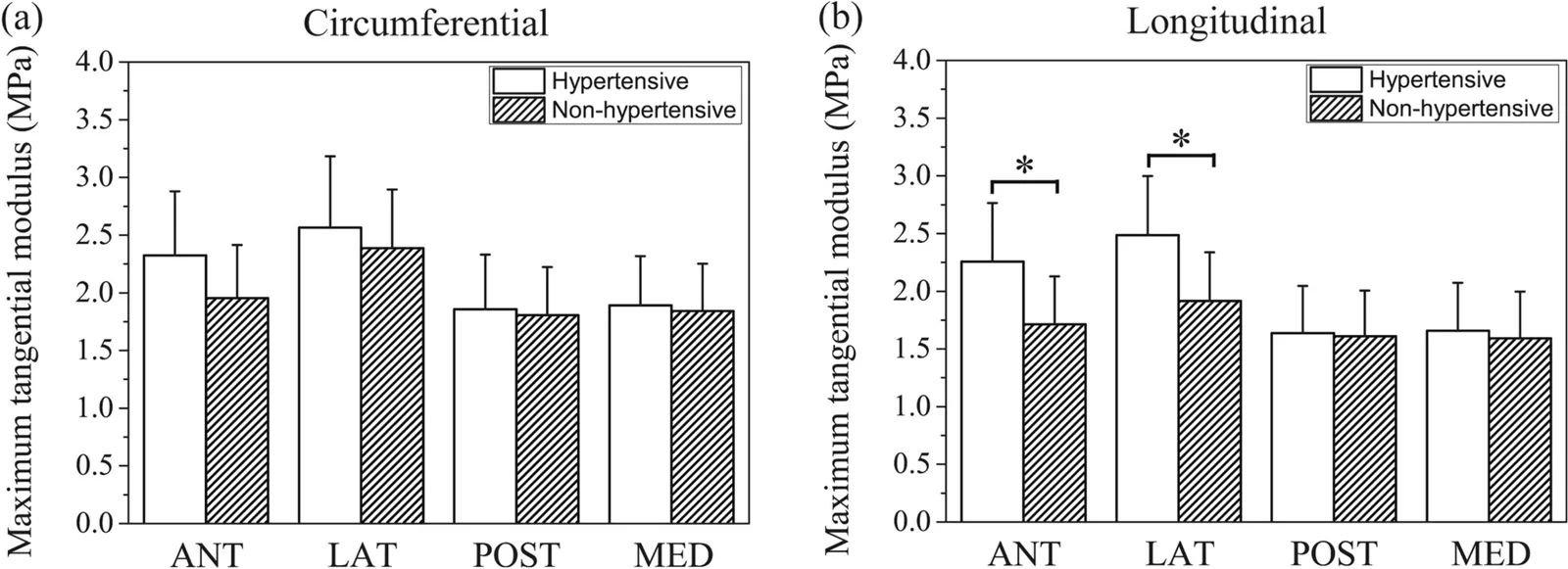
Column plots of regional circumferential and longitudinal maximum tangential modulus (MTM) values in four regions of hypertensive and non-hypertensive BAV-ATAAs. ANT, LAT, POST, and MED denote anterior, lateral, posterior and medial regions, respectively. In the anterior and lateral regions, the longitudinal MTM values of the hypertensive BAV-ATAAs are statistically significantly higher (marked with a star) than those of the non-hypertensive BAV-ATAAs

The averaged circumferential and longitudinal Cauchy stress vs stretch curves (at equibiaxial stresses of *P*_*θθ*_* : P*_*LL*_ = 1 : 1) in four regions of the hypertensive and non-hypertensive BAV-ATAAs are shown in Fig. 3. The regional biaxial mechanical behavior of the BAV-ATAAs was nonlinear and generally anisotropic for both the hypertensive and non-hypertensive groups. The region-specific MTM values of the hypertensive and non-hypertensive BAV-ATAAs are shown in Fig. 4. The relevant data are summarized in Table 3. Under equibiaxial stresses of *P*_*θθ*_* = P*_*LL*_ = 140 kPa, the mean MTM values were highest in the lateral region for both hypertensive and non-hypertensive BAV-ATAAs. There were no significant differences in circumferential MTM values between the hypertensive and non-hypertensive groups (*p* > 0.05). In the anterior and lateral regions, the longitudinal MTM values of the hypertensive BAV-ATAAs were statistically significantly higher than those of the non-hypertensive BAV-ATAAs (2.26 ± 0.51 vs. 1.71 ± 0.42 MPa, *p* = 0.03; 2.49 ± 0.51 vs. 1.92 ± 0.42 MPa, *p* = 0.02).

**Table 3:** Maximum tangential modulus (MTM) values in the circumferential and longitudinal directions for anterior, lateral, posterior, and medial regions of hypertensive and non-hypertensive BAV-ATAAs under equibiaxial stresses

		Aortic stiffness, MTM (MPa)		
		Hypertensive (*n* = 17)	Non-hypertensive (*n* = 8)	*p-*value
Anterior	Circ	2.32 ± 0.56	1.95 ± 0.46	0.17
	Long	2.26 ± 0.51	1.71 ± 0.42	0.03
Lateral	Circ	2.57 ± 0.62	2.39 ± 0.51	0.48
	Long	2.49 ± 0.5	1.92 ± 0.42	0.02
Posterior	Circ	1.86 ± 0.48	1.81 ± 0.42	0.79
	Long	1.64 ± 0.41	1.61 ± 0.39	0.87
Medial	Circ	1.89 ± 0.43	1.84 ± 0.41	0.81
	Long	1.66 ± 0.42	1.59 ± 0.4	0.72

### Material Modeling

**Fig. 5:**
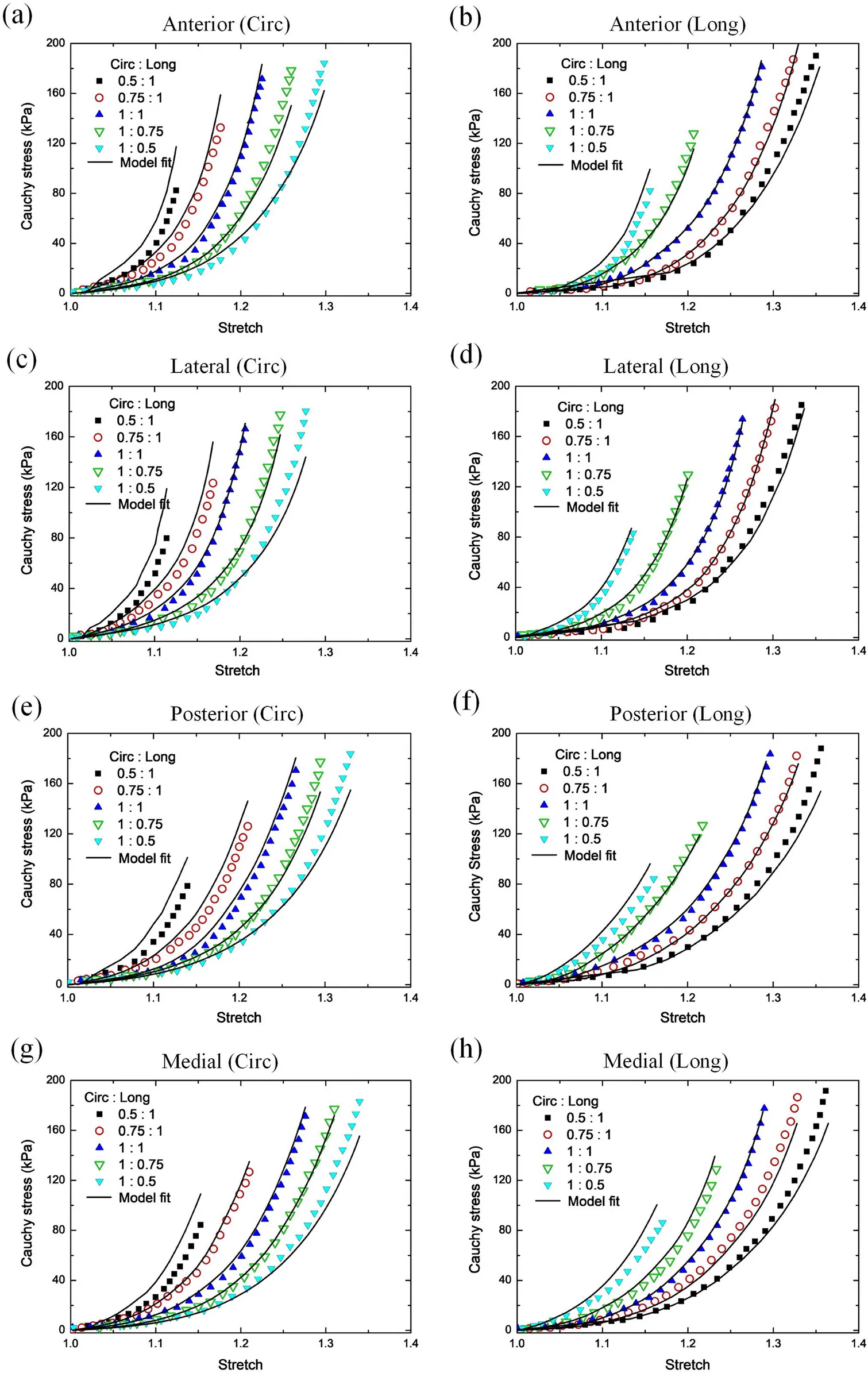
Representative plots of biaxial mechanical data (symbols) with the corresponding model-fitting results (solid curves) from the anterior, lateral, posterior, and medial regions of a non-hypertensive BAV-ATAA (donor age, 59 years). The symbols indicate different ratios of the engineering stresses exerted on the square specimen in the circumferential (Circ) and longitudinal (Long) directions

Figure 5 shows representative plots of regional biaxial experimental data with model-fitting results (as solid curves) of a non-hypertensive BAV-ATAA. The relevant model parameters determined by fitting multi-ratio biaxial data are summarized in Table 4. The ‘goodness of fit’ *R*^2^ values were 0.93 ± 0.03 for the hypertensive group and 0.95 ± 0.02 for the non-hypertensive group. Although the mean values of the model parameter *μ* were higher in the hypertensive patients than in the non-hypertensive patients, no statistically significant differences were observed between two groups in any region (all *p*-values are greater than 0.05). The model parameters *k*_1_ and *k*_2_ in the anterior and lateral regions were significantly larger for the hypertensive BAV-ATAAs than for the non-hypertensive ones (anterior: *k*_1_, *p* = 0.01, *k*_2_, *p* = 0.02; lateral: *k*_1_, *p* < 0.01, *k*_2_, *p* = 0.02). There were no statistically significant differences in the model parameters *φ* and *ρ* between the hypertensive and non-hypertensive groups in any region (all *p*-values are greater than 0.05).

**Table 4:** Model parameters (mean ± SD) for anterior, lateral, posterior, and medial regions of hypertensive and non-hypertensive BAV-ATAAs

	Constitutive Parameters					Goodness of fit
	*μ* (kPa)	*k*1 (kPa)	*k*2	*φ* (°)	*ρ*	*R*^2^
*Anterior*						
H	2.6 ± 0.3	41.3 ± 5.0	3.5 ± 1.5	30.7 ± 5.1	0.21 ± 0.05	0.93 ± 0.03
NH	2.1 ± 0.4	33.8 ± 4.2	1.9 ± 0.7	24.8 ± 4.7	0.19 ± 0.04	0.94 ± 0.03
*Lateral*						
H	3.2 ± 0.7	45.6 ± 6.2	5.4 ± 2.3	29.4 ± 5.5	0.25 ± 0.09	0.92 ± 0.04
NH	3.5 ± 0.6	36.3 ± 4.5	3.2 ± 0.9	27.6 ± 4.2	0.23 ± 0.06	0.95 ± 0.02
*Posterior*						
H	2.0 ± 0.5	37.9 ± 6.2	2.6 ± 1.2	24.3 ± 5.8	0.18 ± 0.06	0.94 ± 0.03
NH	2.4 ± 0.4	32.6 ± 4.0	2.3 ± 1.1	27.5 ± 4.6	0.15 ± 0.03	0.96 ± 0.02
*Medial*						
H	2.3 ± 0.4	32.7 ± 4.5	1.9 ± 0.8	25.8 ± 6.6	0.16 ± 0.03	0.94 ± 0.02
NH	2.5 ± 0.5	29.3 ± 3.8	2.3 ± 1.4	22.7 ± 5.2	0.13 ± 0.05	0.94 ± 0.03

H, hypertensive; NH, non-hypertensive

### Histology

**Fig. 6:**
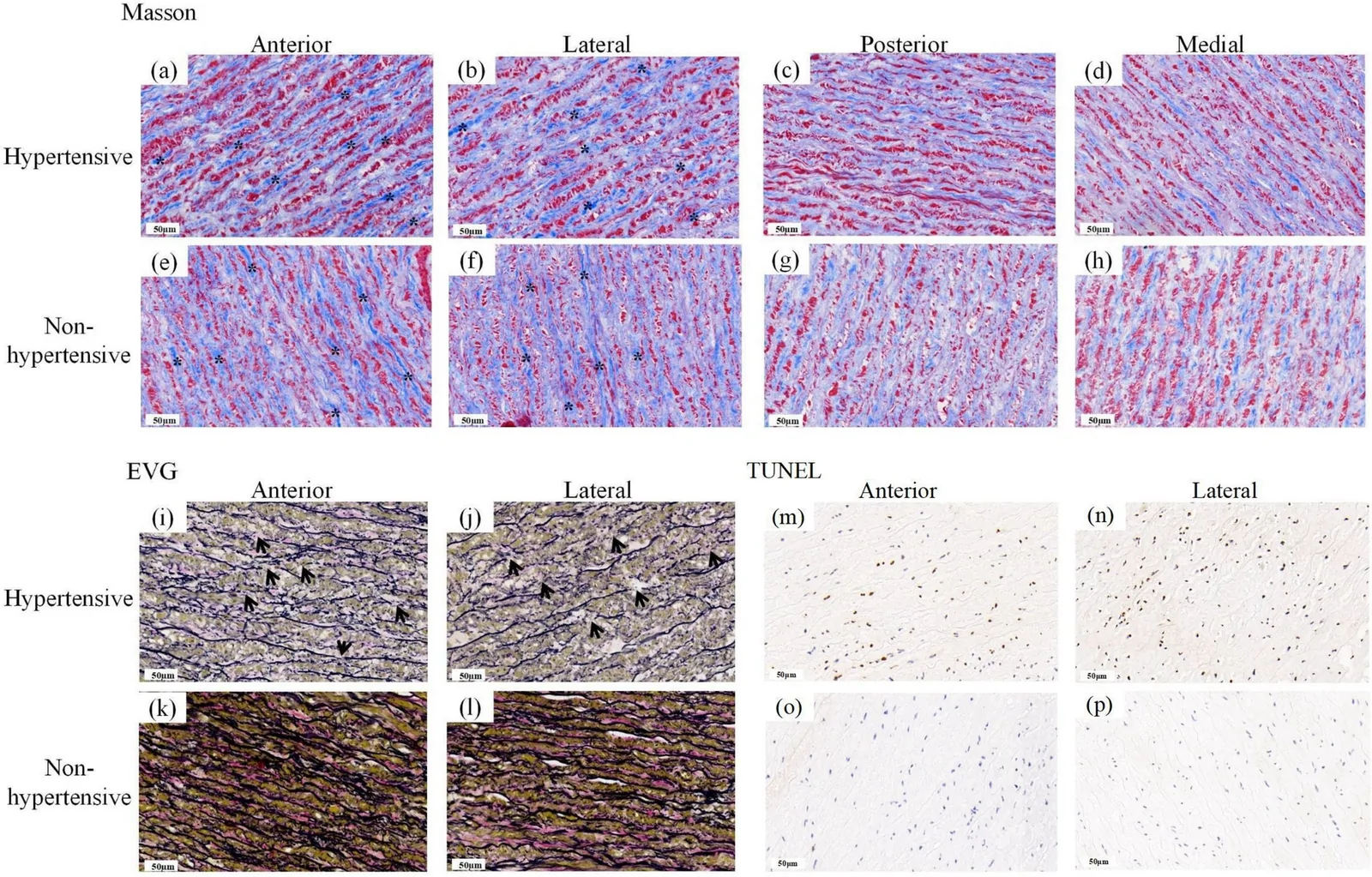
Histological images showing the underlying microstructural features in four regions of hypertensive and non-hypertensive BAV-ATAAs. Collagen fibers stain blue in the Masson staining, whereas elastic fibers stain black to blue/black in the EVG staining. Apoptotic SMCs appear brown after staining with TUNEL. In panels (**a**, **b**) and (**e**, **f**), marked collagen fiber hyperplasia (indicated by asterisks) is observed in the anterior and lateral regions of the hypertensive and non-hypertensive BAV-ATAAs. In the anterior and lateral regions, the laminar structure of the elastic fibers is disrupted in several locations (indicated by arrows) in the hypertensive BAV-ATAAs in panels (**i**, **j**) compared to the non-hypertensive samples in panels (**k**, **l**). SMC death can be observed in the anterior and lateral regions of the hypertensive BAV-ATAAs, see panels (**m**) and (**n**), in contrast to the non-hypertensive BAV-ATAAs in panels (**o**) and (**p**). Original magnification 40 ×

**Fig. 7:**
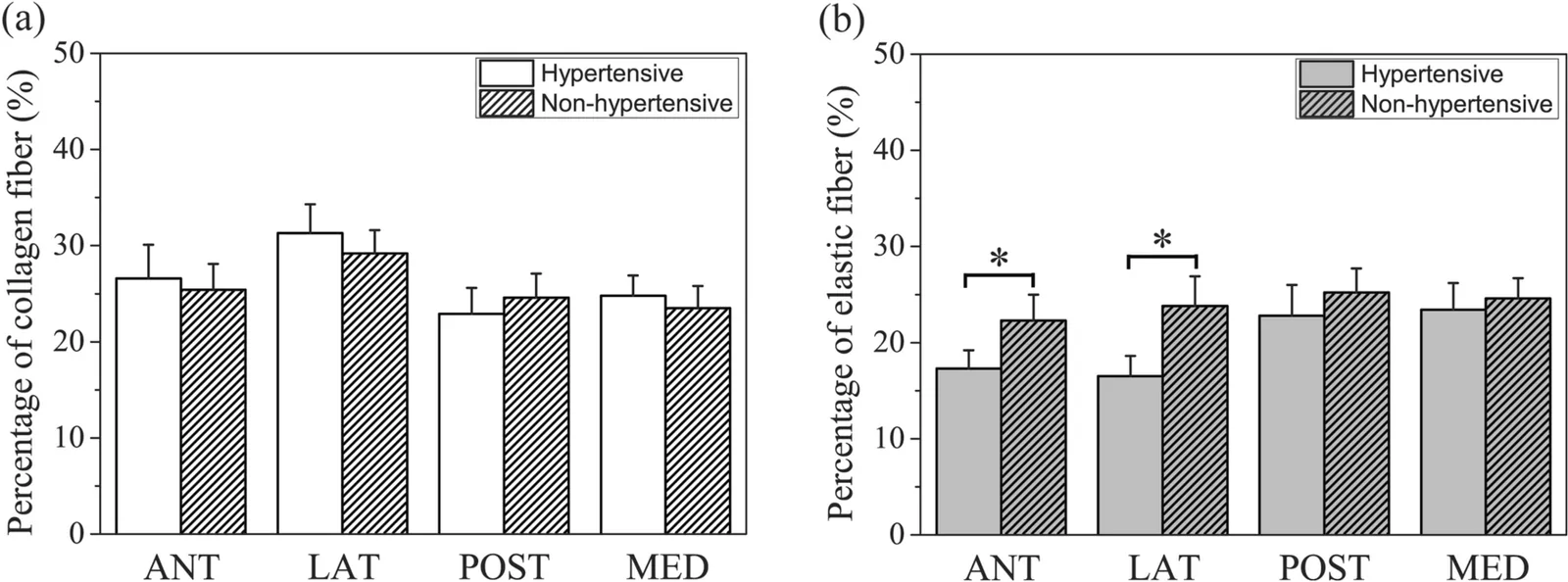
The percentages of (**a**) collagen and (**b**) elastic fibers in four regions of the hypertensive and non-hypertensive BAV-ATAAs. ANT, LAT, POST, and MED denote anterior, lateral, posterior, and medial regions, respectively. Significant differences in the percentages of elastic fibers for the anterior and lateral regions were observed between the hypertensive and non-hypertensive groups

Figure 6 shows representative histological images of four regions of hypertensive and non-hypertensive BAV-ATAAs. Masson staining showed pronounced collagen fiber hyperplasia (marked with asterisks) in the anterior and lateral regions (Fig. 6a, b and e, f) of the hypertensive and non-hypertensive BAV-ATAAs. EVG staining showed that the laminar structure of the elastic fibers was disrupted at several locations in the anterior and lateral regions of the hypertensive BAV-ATAAs (Fig. 6i, j) compared to the non-hypertensive BAV-ATAAs (Fig. 6k, l). Moreover, TUNEL staining showed that the death of smooth muscle cell (SMC) (dark brown) occurred in the anterior and lateral regions of the hypertensive BAV-ATAAs (Fig. 6m, n) in contrast to the non-hypertensive BAV-ATAAs (Fig. 6o, p). The percentages of elastic and collagen fibers in different regions of the hypertensive and non-hypertensive BAV-ATAAs are shown in Fig. 7. There were no statistically significant differences in the percentages of collagen fibers in each region between the hypertensive and non-hypertensive groups (Fig. 7a). As shown in Fig. 7b, the percentages of elastic fibers in the anterior and lateral regions of the hypertensive BAV-ATAAs were statistically significantly lower than those of the non-hypertensive BAV-ATAAs (anterior: 17.3 ± 1.9% vs 22.3 ± 2.7%, *p* = 0.02; lateral: 16.5 ± 2.1% vs. 23.8 ± 3.1%, *p* = 0.01).

### Correlation of Regional Biomechanical Variables with Percentage of Collagen

**Fig. 8:**
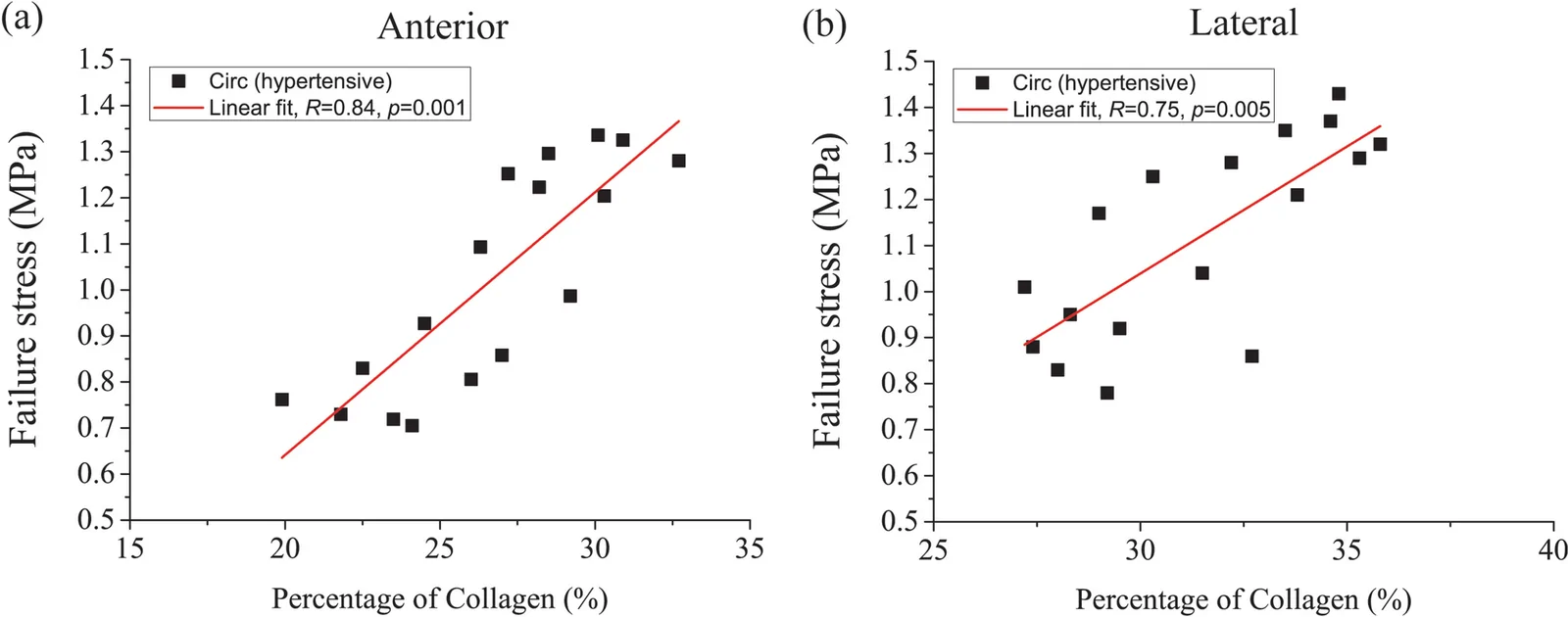
Linear fit to scattered data showing a significant positive correlation between circumferential failure stress and collagen fraction in the (**a**) anterior and (**b**) lateral regions of the hypertensive BAV-ATAAs

As shown in Fig. 8a and b, circumferential failure stresses were significantly increased in the anterior and lateral regions and strongly correlated with collagen content in the hypertensive BAV-ATAAs (anterior: *R* = 0.84, *p* = 0.001; lateral: *R* = 0.75, *p* = 0.005). However, in the non-hypertensive group, no strong correlations between failure stress and collagen content were observed in any region. There was no strong correlation between biaxial mechanical data and collagen content for the hypertensive and non-hypertensive groups.

## Discussion

Many previous studies indicate heterogeneity in the local biomechanical properties of ATAA tissue [1, 5, 7, 21]. Although it is widely accepted that hypertensive BAV-ATAAs exhibit regional differences in their biaxial mechanical behavior [43], a comparison of the regional elastic properties of BAV-ATAAs between hypertensive and non-hypertensive groups is not yet available due to a lack of biomechanical data. Therefore, we thoroughly investigate the failure and biaxial mechanical properties of age-matched hypertensive and non-hypertensive BAV-ATAAs, as well as the underlying microstructural features.

To the authors’ knowledge, this study is the first to provide a complete set of biomechanical data, including circumferential and longitudinal failure stresses, in four regions of both hypertensive and non-hypertensive BAV-ATAAs [9, 13]. Since the tissue strength and elastic properties of aneurysmal ascending aortas decrease with age [28, 29, 40], age-matched hypertensive and non-hypertensive ATAA samples are more suitable for comparison. As shown in Fig. 2 and Table 2, the circumferential failure stresses in the anterior, lateral, and posterior regions are significantly lower in hypertensive BAV-ATAAs than in non-hypertensive BAV-ATAAs. This suggests that hypertension, as an important pathological factor, may have a significant impact on the tensile strength of BAV-ATAA tissue, especially in the circumferential direction. In contrast, no statistically significant differences in longitudinal failure stresses were found between hypertensive and non-hypertensive groups in the lateral, posterior, and medial regions. These findings are basically consistent with a previous study [13], which showed significant differences in circumferential peak stresses between normotensive and hypertensive groups for the anterior region. Interestingly, the circumferential and longitudinal peak stresses in the anterior region of the ATAAs reported by Ferrara et al. [13] in hypertensive patients are similar to the values we measured in the lateral region. It should also be emphasized that the tensile strength of BAV-ATAAs is higher in both directions than that of ATAAs with a tricuspid aortic valve (TAV) [29]. In the present study, the failure stress is defined as the Cauchy stress value at the maximum force during the UT test (Sherifova et al. 2019). Compared with previous experimental investigations of the failure properties of ATAAs with different aortic valve morphologies [9, 20, 23, 29], our failure stress results are within the same range of the biomechanical data reported in these studies. In addition to the UT test, bulge inflation test was also performed to investigate the rupture of ATAAs under multiaxial extension [11].

Although the biaxial mechanical properties of ATAAs have been extensively investigated [2], Cosentino et al. 2023; [21, 36, 37, 42, 43], hypertension-induced changes in aortic stiffness are rarely reported in the literature [21, 28]. As shown in Fig. 3, the mechanical behavior in four regions of hypertensive and non-hypertensive BAV-ATAAs under biaxial loading is nonlinear. In particular, mechanical anisotropy appear to be observed in some specimens from the anterior and lateral regions. As shown in Fig. A1, the shear stress components in the circumferential and longitudinal directions are significantly lower than the first Piola-Kirchhoff stresses with increasing loading duration. This suggests that the shear deformation can be neglected in our biaxial tests. Since the shear is assumed to be zero, the deformation gradient components *F*_11_ and *F*_22_ are computed as the ratio of current gauge length to the initial gauge length in the circumferential and longitudinal directions, respectively. At the given stress rate during biaxial extension, *F*_11_ and *F*_22_ increase nonlinearly with the loading duration (Fig. A2). Both hypertensive and non-hypertensive ATAA tissues exhibit mechanical anisotropy with increasing loading duration. It should be noted that planar biaxial experiments cannot theoretically fully characterize the anisotropic elastic properties of soft tissue unless certain assumptions are made [17].

At equibiaxial stresses (*P*_*θθ*_* = P*_*LL*_ = 140 kPa), the results suggest that the hypertensive BAV-ATAAs have significantly higher longitudinal tissue stiffness than the non-hypertensive BAV-ATAAs in the anterior and lateral regions (Fig. 4 and Table 3). However, there were no significant differences in circumferential aortic stiffness between hypertensive and non-hypertensive groups in any region. This indicates that tissue stiffening primarily occurs in the longitudinal direction of the hypertensive BAV-ATAAs. As the outer curvature of the aneurysmal ascending aorta, the anterior and lateral regions are therefore considered special sites with limited elastic properties for the hypertensive BAV-ATAAs. Our findings differ from Pham et al. [28], who suggested no significant difference in stiffness between hypertensive and non-hypertensive BAV patients. However, in patients without BAV, they found significantly higher longitudinal stiffness in the hypertensive groups than in the non-hypertensive groups. In the above-mentioned study, a very small number of patients with BAV and hypertension were collected for biaxial mechanical testing and comparison. More importantly, regional aortic tissue sampling was not considered. Ferrara et al. [13] also suggested that there are no significant differences in the high elastic modulus of ATAAs between normotensive and hypertensive patients. Note that their results were from a uniaxial tensile test. In a similar study, Lin et al. [21] measured the aortic stiffness before failure of 100 ATAA samples and found that stiffness was not affected by hypertension. However, the valve types in the groups with and without hypertension include both BAV and TAV. Moreover, it is unknown whether the two groups are age-matched. Therefore, selecting different patient cohorts to explore the effects of hypertension may lead to discrepancies. From a biomechanical perspective, the mean aortic stiffness of the TAV-ATAAs, measured by Cosentino et al. (2023), is lower in all regions than that of the hypertensive BAV-ATAAs at a stretch of 1.1.

The present study also highlights the regional heterogeneity in the biomechanical properties of hypertensive and non-hypertensive ATAAs. In particular, the lateral region exhibits higher stiffness compared to other regions, which is consistent with findings from previous studies (Cosentino et al. 2023; [10, 20, 36]). In BAV patients, regional variations in the local elastic properties of ATAAs may be attributed to valve-related hemodynamics [8, 44]. For example, Youssefi et al. [44] suggested that BAV-induced eccentric flow may lead to increased wall shear stress (WSS) on the wall of the ascending aorta. Hemodynamic analyses by Condemi et al. [6] showed that in BAV patients the peak time-averaged wall shear stress (TAWSS) occurs in the anterior region of ATAAs. The authors also found a statistically (highly) significant positive correlation between rupture stress and TAWSS. Thus, a relatively low TAWSS could correlate significantly with reduced rupture properties in ATAAs. Our results support this hypothesis, as the failure stresses of BAV-ATAAs are higher in the anterior region than in the posterior and medial regions in both patient groups with and without hypertension. In particular, our measured failure stress values are in the same range as the Cauchy stresses at rupture in the aforementioned study, i.e., between 0.5 and 1.6 MPa. A previous study (McClarthy et al. 2021) found no statistically significant correlation between TAWSS and the elastic modulus of ATAA tissue at 10% strain. However, the elastic modulus represents tissue stiffness in areas of relatively low loading, which is significantly lower than the regional aortic stiffness we determined.

The material model we chose is appropriate for characterizing the biaxial mechanical behavior in four regions of hypertensive and non-hypertensive BAV-ATAAs (Fig. 5). In the low-load range, i.e., where the matrix material is primarily active, the determined parameter *μ* suggests that there is no significant difference in tissue stiffness between hypertensive and non-hypertensive groups in any region (Table 4). In the high-load range, where collagen fibers are recruited, the parameters *k*_1_ and *k*_2_ indicate that the hypertensive BAV-ATAAs exhibit significantly higher tissue stiffness in the anterior and lateral regions than the non-hypertensive samples. The parameter *φ* likely indicates that there are no significant differences in the orientation of the major collagen fibers in any region between the two groups. Note, however, that *φ* is phenomenologically determined in the current study. Both histological measurements and multi-photon imaging analyses can be used to determine the collagen fiber angle in material modeling of aneurysmal and healthy thoracic aortas (Cosentino et al. 2023; [33], Schriefl et al. 2012). Compared with a recent study (Cosentino et al. 2023), our derived mean collagen fiber angles in four regions of the hypertensive and non-hypertensive ATAAs are within the same range as their fiber angles measured by multi-photon imaging. The parameter *ρ* shows that the mechanical anisotropy of the ATAA tissue does not differ significantly between the hypertensive and non-hypertensive groups. In the present study ten curves from five biaxial protocols are fitted simultaneously with a set of material parameters. Due to this fitting algorithm, some obvious deviations are observed between the fitted curves and the original experimental data, e.g., in the stress ratio of 0.5: 1.

As shown in Fig. 6, histological analysis suggests that remarkable collagen fiber hyperplasia is present in the anterior and lateral regions of both hypertensive and non-hypertensive BAV-ATAAs compared to the posterior and medial regions. However, in contrast to the non-hypertensive group, elastic fiber disruption and SMC death in the hypertensive group occur primarily at various locations in the anterior and lateral regions. These microstructural alterations may lead to lower failure stress and impaired elastic properties of BAV-ATAAs in hypertensive patients. Previous studies have investigated the relationship between WSS and the histopathology of elastic fibers. They suggested that elevated WSS may further accelerate elastic fiber thinning and wall degradation in BAV patients [3, 15, 32]. In particular, in patients with normal WSS, elastic fibers are thinner and WSS is higher along the greater curvature than along the lesser curvature. These results are in line with our histological observations, which show thinning and disruption of elastic fibers at multiple sites in the lateral region of BAV-ATAAs. Semi-quantitative measurements show that the proportion of elastic fibers is significantly reduced in the anterior and lateral regions of hypertensive BAV-ATAAs compared to non-hypertensive BAV-ATAAs (Fig. 7). As important load-bearing components, changes in collagen and elastic fiber content may significantly alter the nonlinear mechanical behavior of the aortic wall and have substantial effects on failure behavior and aortic stiffness [34, 42]. In addition, the outer curvature segment of the ATAA wall appears to be highly susceptible to microstructural changes caused by both hypertension and BAV-related hemodynamics.

Among all correlations, circumferential failure stresses in the anterior and lateral regions are significantly increased and strongly correlate with collagen content in the hypertensive group (Fig. 8). As mentioned previously, collagen fibers play a key role in preventing tissue failure during tensile tests. Due to the disruption of elastic fibers in the anterior and lateral regions, the failure stress of hypertensive ATAA tissue is likely more dependent on collagen content. Although Pichamuthu et al. [29] suggested a moderate correlation between collagen content and tensile strength for the TAV-ATAAs and not for the BAV type, the hypertension effect and regional differences were not considered in the biomechanical analysis.

Some limitations of the present study are also summarized. First, our sample size is relatively small, especially for the ATAA samples from age-matched, non-hypertensive BAV patients. In clinical practice, most BAV-ATAAs are acquired from patients with a history of hypertension. Therefore, a larger sample size is needed to further investigate the pathological effect on the regional biomechanical properties of the dilated ascending aortas. Second, changes in regional hemodynamics of hypertensive and non-hypertensive BAV-ATAAs have not been previously investigated and compared. Since valve-related hemodynamics is a contributing factor to the deterioration of the elastic properties of the ATAA wall, it would be very useful to establish correlations between hemodynamic parameters and regional biomechanical variables [6], McClarthy et al. 2021).

## Conclusion

Despite the above limitations, our biomechanical analyses show that the regional elastic properties of hypertensive BAV-ATAAs are more severely impaired than those of non-hypertensive samples. In the anterior and lateral regions, hypertensive BAV-ATAAs exhibit significantly lower circumferential failure stress and higher longitudinal aortic stiffness than non-hypertensive BAV-ATAAs. As an important pathological factor, hypertension therefore significantly affects the tensile strength and tissue stiffness of BAV-ATAAs. The vast majority of the included patients with hypertension (15/17) had grade 2 or higher hypertension prior to admission. Changes in biomechanical properties suggest that patients with a BAV and grade 2 or higher hypertension (i.e., systolic blood pressure ≥ 160 mmHg) have a higher risk of ATAA rupture in the hospital than patients with BAV without hypertension.

Furthermore, elastic fiber disruptions and collagen fiber hyperplasia appear to be observed in the anterior and lateral regions of hypertensive BAV patients, confirming that the outer curvature of the ATAA wall is subject to hypertension-induced aortic remodeling. Notably, the circumferential failure stress in these regions strongly correlates with the collagen content in the hypertensive group. Using the regional material parameters determined in the present study, further finite element simulations will be performed to investigate differences in the wall stress distribution of the dilated ascending aorta between hypertensive and non-hypertensive BAV patients [14].
